# Enhancing the Resistance to Shear Instability in Cu/Zr Nanolaminates Through Amorphous Interfacial Layer

**DOI:** 10.3390/nano15171323

**Published:** 2025-08-28

**Authors:** Feihu Chen, Feng Qin

**Affiliations:** 1 College of Mechanical and Electrical Engineering, Central South University, Changsha 410083, China; cfhazn@163.com; 2 Chengdu Tool Research Institute Co., Ltd., Chengdu 610051, China

**Keywords:** metallic nanolaminates, crystalline/amorphous interface, indentation, shear band

## Abstract

Metallic nanolaminates generally show ultra-high strength but low ductility due to their vulnerability to shear instability during deformation. Herein, we report the simultaneous enhancement in hardness (by 11.9%) and suppression of shear instability in a 10 nm Cu/Zr nanolaminate, achieved by introducing a nanoscale Cu_63_Zr_37_ amorphous interfacial layer (AIL) between the crystalline Cu and Zr layers via magnetron sputtering. The effect of AIL and its thickness (*h*) (*h* = 2, 5, and 10 nm) on the hardness and shear instability behavior was explored using nano- and micro-indentation tests. An abnormal increase in hardness occurs at *h* = 2 nm when *h* is decreased from 10 to 2 nm, deviating from the prediction of the rule of mixtures. This abnormal strengthening is attributed to thinner AIL, which induces an increased density of crystalline/amorphous interfaces, thereby generating a pronounced interface strengthening effect. The micro-indentation results show that shear banding was suppressed in the nanolaminate with AIL, as evidenced by fewer shear bands as compared to its homogeneous counterpart. This enhanced resistance to shear instability may originate from the crystalline/amorphous interface that provides more sites for dislocation nucleation, emission, and annihilation. Furthermore, two distinct shear banding modes were observed in the nanolaminate with AIL; i.e., a cutting-like shear banding emerged at *h* = 10 nm, whereas a kinking-like shear banding occurred at *h* = 2 nm. The potential mechanism of the AIL-thickness-dependent shear banding was analyzed based on the crack propagation model of the Griffith criterion. This study provides a comprehensive insight into the strengthening and tunable shear instability of super-nano metallic laminates by AIL.

## 1. Introduction

Metallic nanolaminates have attracted extensive scientific interest in materials science because of their exceptional mechanical properties [1,2,3], such as ultra-high strength [4,5,6,7], excellent thermal stability [8,9,10,11], and good radiation damage tolerance [12,13,14,15]. However, nanolaminates generally exhibit poor strain hardening due to the extremely confined dislocation activity in the nanoscale interface space, making them prone to premature strain localization-induced failure. For example, uniaxial tensile tests on Cu/Zr, Cu/Nb, and Cu/Ru nanolaminates revealed an extremely low elongation (less than 4%) [16,17,18]. Shear banding is a typical strain localization-induced plastic instability that is prevalent in the plastic deformation of nanolaminates, nanocrystalline metals, and amorphous alloys [19,20,21,22,23]. For example, micro- and nano-indentation tests on the Cu/Au, Cu/Cr, Cu/Nb, Cu/Ta, and Cu/Zr nanolaminates demonstrate that profuse circular shear bands (SBs) consistently emerge at the indentation surface when the individual layer thickness of nanolaminates is reduced to several tens of nanometers [19,20,23,24,25,26,27]. The formation of circular SBs in these nanolaminates was attributed to the grain boundary-mediated movement (accompanied by grain rotation) due to limited dislocation activity. Further, when subject to micropillar compression (approximate uniaxial stress state), nanolaminates also exhibit several typical plastic instabilities, such as shear banding mediated by interface rotation and sliding [28,29] and interface failure induced by the incompatible deformation between soft and hard layers [30,31].

To suppress the shear instability of nanolaminates, two typical strategies have been proposed by researchers, i.e., topological structure design and interface engineering [32,33,34,35,36,37]. The first strategy is rooted in manipulating the interface density or interface space in a non-uniform distribution, such as bimodal, gradient, and sandwich architectures. For example, a 4 nm Cu/Nb nanolaminate showed profuse shear cracks after the rolling of a 4% strain [38], while shear cracks were completely suppressed in a bimodal Cu/Nb nanolaminate composed of alternating 40 nm Cu/Nb and 4 nm Cu/Nb bilayers after the rolling of a 30% strain, as reported by Wynn et al. [32]. Additionally, the bimodal, gradient, and heterogeneous Cu/Zr nanolaminates designed by Li et al. [33,34] also show effectiveness in suppressing SB formation during micropillar compression. Moreover, the molecular dynamics simulations conducted by Wang et al. [39] indicated that a compatible deformation between the 24 nm Cu (soft phase) and Ni (hard phase) layers in Cu/Ni nanolaminates can be achieved through a heterostructural design, i.e., introducing a 2.5 nm Cu/Ni bilayer on both sides of the 24 nm Cu and Ni layers.

As for the second strategy, a common and effective method is to induce an amorphous phase into the interface region between the constituents of nanolaminates. Notably, the initial crystalline/crystalline (C/C) interface of nanolaminates is thus replaced by the crystalline/amorphous (C/A) interface. A pioneering work was conducted by Wang et al. [40], who fabricated a 35 nm Cu/5 nm CuZr amorphous nanolaminate, simultaneously achieving a strength of 1.1 GPa and a tensile ductility of 13.8%, which is significantly higher than that of conventional C/C nanolaminates. Donohue et al. [41] performed cold rolling experiments on 90 nm Cu/10 nm PdSi amorphous nanolaminate and observed that the constituent layer was uniformly thinned by over 75%, completely suppressing the generation of SBs in the amorphous layer. Additionally, the micropillar compression experiments on Zr/CuZr amorphous nanolaminate, carried out by Liu et al. [42], revealed that the amorphous layers achieved a plastic strain of over 50% at room temperature. This is attributed to the constraint of the crystalline layer on the amorphous one, which can block and absorb the incident SBs from the amorphous layer. In addition, Kim and Fan et al. [43,44] demonstrated that the highly localized deformation in the amorphous phase can be suppressed by tuning the thickness of the amorphous layers in Cu/CuZr nanolaminate, thereby improving the plastic deformability. The principle is to restrict the aggregation of shear transition zones (STZ) by limiting the geometric size of the amorphous phase, thereby suppressing the nucleation of SB in the amorphous layer. Interestingly, Chen et al. [36,37] introduced a 3D interface into the Cu/Nb nanolaminate, which significantly improves the yield strength (~50% enhancement) and suppresses shear instability. This is attributed to the fact that the 3D interface can reduce stress concentration caused by dislocation pileup and prevent shear localization. Moreover, Misra et al. [45] performed molecular dynamics (MD) simulations on Cu/CuZr nanolaminates, revealing that the C/A interface acts as a sink for dislocations in the crystalline layer via interfacial shear mechanisms. This process effectively suppresses the formation of dislocation networks and the nucleation of SBs, thereby enhancing the ductility of the material.

Although extensive studies have explored the influence of the AIL on the mechanical properties of nanolaminates, previous studies were focused on the deformation of nanolaminates with AIL under a uniaxial stress state (e.g., micropillar compression and uniaxial tensile tests). The effect of the AIL on the shear instability behavior of nanolaminates under complex stress states is rarely reported, especially for nanolaminates with an extremely small layer thickness (below 10 nm).

In this work, 10 nm Cu/Zr nanolaminates with different AIL thicknesses, i.e., 2 nm, 5 nm, and 10 nm, respectively, were prepared using magnetron sputtering. The AIL-dependent hardness and shear banding behavior of the nanolaminates were systematically studied by nano- and micro-indentation tests, respectively. As a distinction to the existing studies, which focus on the influence of the amorphous phase on the mechanical properties of the single homogeneous phase, e.g., soft Cu [22,30,40,46], the task of this study aims to clarify how the AIL and its thickness variations systematically modulate the mechanical performance of heterogeneous metallic nanolaminates with hard and soft domains. The results of this study show that the hardness of the Cu/Zr nanolaminates with AIL exhibits a monotonic decrease as AIL thickness (*h*) reduces from 10 nm to 5 nm, which can be well described by the rule of mixtures. Interestingly, two types of shear banding were first observed in the newly designed Cu/Zr nanolaminates with AIL—i.e., cutting-like shear banding formed in nanolaminates with 10 nm AIL and kinking-like shear banding formed in nanolaminates with 2 nm AIL—showing significantly different mechanisms from the previous studies that only reported the former shear banding mode at large individual layer thickness (>20 nm) [22,46]. These findings uncover the tunable shear instability of super-nano metallic laminates by AIL.

## 2. Materials and Methods

### 2.1. Sample Preparation

**Figure 1:**
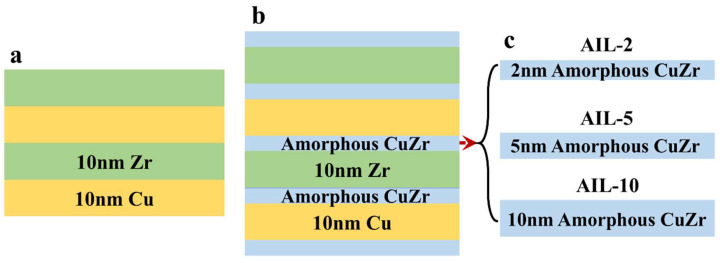
Schematic of (**a**) homogeneous and (**b**,**c**) designed nanolaminates with AIL structures. Three Cu/Zr nanolaminates with AIL were designed—AIL-2, AIL-5, and AIL-10—where the number represents the thickness of the amorphous interlayer.

The Cu/Zr nanolaminates were deposited on a Si (100) substrate with a natural oxide layer by direct current magnetron sputtering (PTL6S PVD system) at room temperature. Si wafer was selected as the substrate due to its atomic-level smooth interface, low thermal expansion coefficient, high intrinsic hardness, and excellent conductivity. These properties promote uniform film growth, achieve a sharp interface of the films, minimize defect formation, alleviate thermal stress-induced cracking, and enable a wide applicability for mechanical testing and microstructure characterization in electron microscopy. The purity of the Cu and Zr targets was 99.99% and 99.9%, respectively. The base pressure of the vacuum chamber was 5 × 10^−5^ Pa. An Ar pressure of 0.3 Pa was used during deposition. Before the deposition of the film, Cu and Zr targets were sputtered at a power of 100 W for one minute to clean the surface oxide. During the deposition, the substrate was maintained at a rotation frequency of 20 rpm to ensure the uniformity of the film. Three Cu/Zr nanolaminates with different AIL thicknesses were prepared, i.e., 2 nm, 5 nm, and 10 nm, as shown in Figure 1b,c, which were designated AIL-2, AIL-5, and AIL-10, respectively. The AIL was deposited on both sides of each crystalline constituent layer of the 10 nm homogeneous Cu/Zr nanolaminate (Figure 1). The corresponding volume fractions of the AIL in samples AIL-2, AIL-5, and AIL-10 were 16.7%, 33.3%, and 50%, respectively. Among all samples, the amorphous CuZr layer was deposited both as the first layer on the Si substrate and as the cap layer. When depositing the Cu and Zr constituent layers, the power used for Cu and Zr targets was 70 W and 120 W, respectively, resulting in a deposition rate of 0.2224 nm/s for Cu and 0.1558 nm/s for Zr. The deposition times for the 10 nm Cu and Zr layers were 45 s and 62 s, respectively. The amorphous CuZr layer was deposited by co-sputtering the Cu and Zr targets. The target alloy composition is Cu_60_Zr_40_ (atomic fraction). The powers used for Cu and Zr targets in the co-sputtering process were 37 W and 120 W, respectively, resulting in a deposition rate of 0.2734 nm/s for the amorphous Cu_60_Zr_40_ layer. The AIL thickness was controlled by tuning the co-sputtering time based on the measurement of the co-sputtering rate for Cu and Zr, i.e., 7 s for sample AIL-2, 18 s for sample AIL-5, and 37 s for sample AIL-10. The total thickness of the Cu/Zr nanolaminates with AIL was ~1.2 μm. Homogeneous Cu/Zr nanolaminates with an individual layer thickness of 10 nm and monolayer amorphous CuZr films with a total thickness of 1.2 μm were also deposited for comparison.

### 2.2. Microstructural Characterization

The crystallographic orientation of the as-prepared films was characterized by X-ray diffraction (XRD, Bruker Advance D8, Bruker, Billerica, MA, USA), and the scanning angle (2θ) range was 20–60°. The cross-sectional microstructures and composition of all samples were examined using a transmission electron microscope (TEM, Talos F200X G2, Thermo Fisher Scientific Inc., Shanghai, China). The cross-sectional TEM lamellas of the as-deposited and indentation-induced nanolaminates were prepared using focused ion beam (FIB) (FEI, Helios Nanolab 600i, and Helios 5CX, Thermo Fisher Scientific Inc., Shanghai, China) milling.

### 2.3. Nano-Indentation and Micro-Indentation Tests

The hardness and elastic modulus of nanolaminates were measured by nano-indentation (Agilent G200, KLA, Chandler, AZ, USA, Berkovich tip with a radius of ~50 nm) under the continuous stiffness measurement (CSM) mode, where the hardness/modulus-displacement curves could be obtained continuously. A constant strain rate of 0.05 s^−1^ was applied in testing. The thermal drift rate was 0.1 nm/s and corrected automatically during testing. The maximum indentation depth was 400 nm. Five indentations were performed for each sample to obtain the average hardness and standard deviation. In addition, micro-indentation tests were performed (Shimadzu HMV-G20ST, Shimadzu, Kyoto, Japan) at room temperature to investigate the shear banding behavior of the nanolaminates. The applied loads were 250 mN and 500 mN with a holding time of 5 s, and each load test was repeated five times in all samples. To avoid the influence of the previous micro-indentation-induced deformation on the next indentation test, the spacing between each micro-indent was greater than 50 μm. The surface morphology induced by nano- and micro-indentations was characterized by scanning electron microscopy (SEM, TESCAN MIRA3, TESCAN, Brno, Czech Republic). After nano-indentation tests, the TEM lamellar of the indent was prepared using a focused ion beam (FIB, Helios 600i).

## 3. Results and Discussion

### 3.1. Microstructure of the As-Deposited Cu/Zr Nanolaminates with AIL

**Figure 2:**
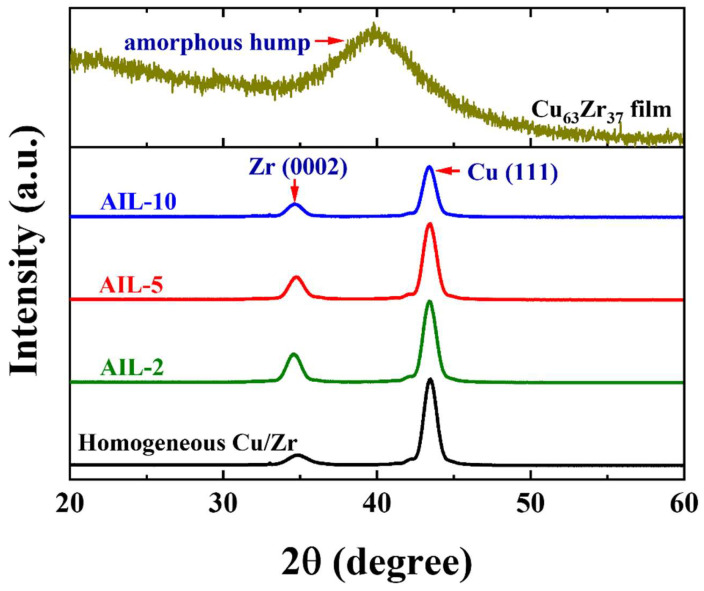
XRD patterns of the Cu/Zr nanolaminates with AIL (*h* = 2 nm, 5 nm, and 10 nm), the homogeneous Cu/Zr nanolaminates, and the monolayer amorphous CuZr film. All AIL nanolaminates show a strong Zr (0002) and Cu (111) texture, which is consistent with that detected in the homogeneous Cu/Zr nanolaminates, and the amorphous CuZr film shows a typical amorphous hump.

Figure 2 shows the XRD patterns of the as-deposited nanolaminates with AIL (AIL-2, AIL-5, and AIL-10), the homogeneous Cu/Zr nanolaminate, and the monolayer amorphous CuZr films. It can be observed that the monolayer CuZr film exhibits typical amorphous characteristics with a wide hump diffraction. The diffraction angle for the amorphous hump in the Cu_63_Zr_37_ film is approximately 40°, which is similar to CuZr, as reported by Sun et al. [47]. These three Cu/Zr nanolaminates with AIL all exhibit strong Zr (0002) and Cu (111) textures, and the intensities of the Zr (0002) and Cu (111) peaks in the three samples decreased with the increase of the AIL thickness. It can clearly be seen that the peaks of Zr (0002) and Cu (111) in the samples with AIL are approximately 34.5° and 43°, respectively, which is consistent with that of homogeneous Cu/Zr samples. This indicates that the introduction of CuZr AIL into the homogeneous Cu/Zr nanolaminates does not significantly alter the crystallographic orientation of the crystalline constituent layers.

**Figure 3:**
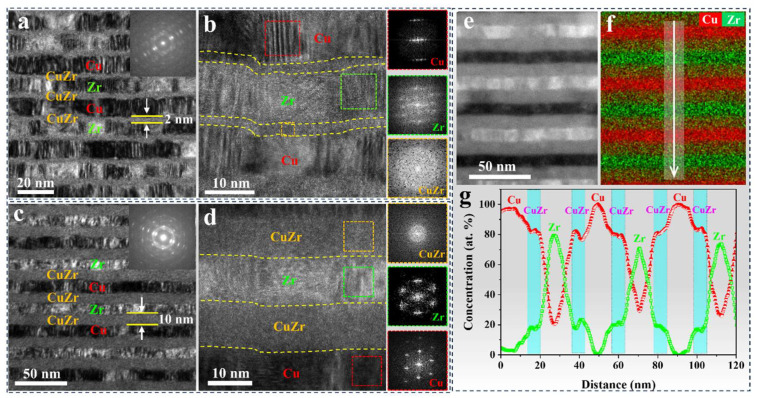
Cross-sectional TEM micrographs of the as-deposited Cu/Zr nanolaminates with AIL. (**a**,**c**) are representative bright-field TEM images of samples AIL-2 and AIL-10, respectively. The insets are the corresponding SAED patterns. The HRTEM images of the AIL-2 (**b**) and AIL-10 (**d**) samples show the clear interfacial structures between the crystalline and the amorphous CuZr layers. The insets in (**b**,**d**) are the corresponding FFTs. (**e**,**f**) are HAADF-STEM images and the corresponding EDS mapping of AIL-10. (**g**) EDS line scanning results along the direction of the white arrow in (**f**), exhibiting a clearly modulated structure and the concentration of Cu and Zr elements.

The cross-sectional microstructure of the as-deposited samples with AIL was characterized using TEM, as illustrated in Figure 3. The representative bright-field TEM images of AIL-2 (Figure 3a) and AIL-10 (Figure 3c) show the modulation layer structure with a clear interface, and both sides of all the crystalline Cu and Zr layers are covered by amorphous CuZr layers. The Cu and Zr layers of the samples exhibit a typical columnar polycrystalline structure along the film deposition direction. The corresponding SADP patterns inserted in Figure 3a,c show a sharp bright spot of Cu (111) and Zr (0002), as well as the amorphous halo ring, which is consistent with the XRD results (Figure 2). The actual thicknesses of the crystalline Cu, Zr, and amorphous CuZr layer in the AIL-2 and AIL-10 samples, measured by TEM, are summarized in Table 1. It can be seen that the actual layer thickness of each sample is consistent with the designed one. The high-resolution TEM (HRTEM) images of samples AIL-2 and AIL-10 are given in Figure 3b,d, and reveal the distinguishable interface of the C/A nanolaminates without intermixing. The fast Fourier transforms (FFT) patterns of the frame position in the insets of Figure 3b,d indicate FCC and HCP orientation in crystalline Cu and Zr layers, respectively, which is in contrast with the halo ring pattern detected in amorphous CuZr layers. High-angle annular dark-field scanning TEM (HAADF-STEM) also shows modulated layered structures of sample AIL-10, as shown in Figure 3e. The corresponding EDS mapping (Figure 3f) indicates a CuZr interlayer between the Cu and Zr layers, with the uniform distribution of Cu and Zr elements. Figure 3g shows the line scan result of Figure 3f along the white arrow direction, indicating that the sample has a chemically alternating layered structure composed of alternating stacked Cu layers, CuZr layers, and Zr layers.

### 3.2. The Hardness of Cu/Zr Nanolaminates with AIL

**Figure 4:**
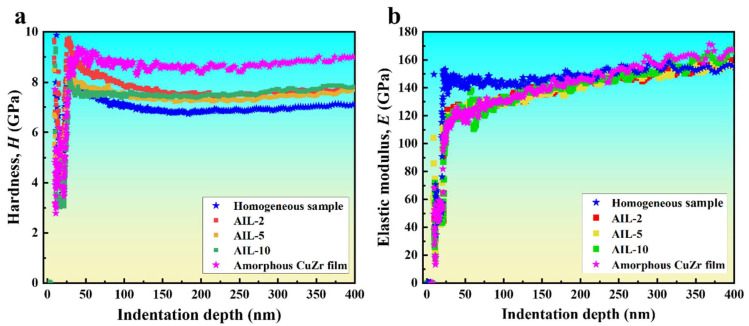
Representative (**a**) nano-hardness and (**b**) elastic modulus as functions of indentation depth for Cu/Zr nanolaminates with AIL (i.e., AIL-2, AIL-5, and AIL-10). The corresponding data for 10 nm homogeneous Cu/Zr and monolayer amorphous CuZr films are included for comparison.

Figure 4a,b show the hardness and elastic modulus of the nanolaminate with AIL, the homogeneous Cu/Zr nanolaminate, and monolayer amorphous CuZr films as functions of indentation depth, respectively. The hardness curves of all samples exhibit similar patterns, i.e., when the indentation depth is relatively small (<50 nm), the hardness decreases rapidly with increasing depth due to the size effect of the indentation. As the penetration depth is further increased, the hardness gradually reaches a plateau value without significantly changing with increasing penetration depth. This shows that the hardness measurement of soft film is negligibly influenced by the hard Si substrate [48]. Thus, the average plateau value from hardness–indentation depth curves (i.e., 150–250 nm) can be adopted to be the intrinsic hardness of the nanolaminates [48]. In contrast, the modulus of all samples increases slowly with indentation depth because of the effect of the Si substrate with higher stiffness. The elastic modulus was adopted from the data below a depth of 10% of the total film thickness, i.e., 50–120 nm, where the substrate effect is insignificant at this depth. The average hardness and modulus measured from five repeated tests were taken as the hardness and elastic modulus for each sample. The measured hardness and elastic modulus of the nanolaminates with AIL are listed in Table 2. It can be observed that the hardness of the samples with AIL decreases and then increases as the thickness of the AIL decreases from 10 nm to 2 nm. The elastic modulus exhibits insignificant variation across composites with amorphous interlayers of varying thickness.

**Figure 5:**
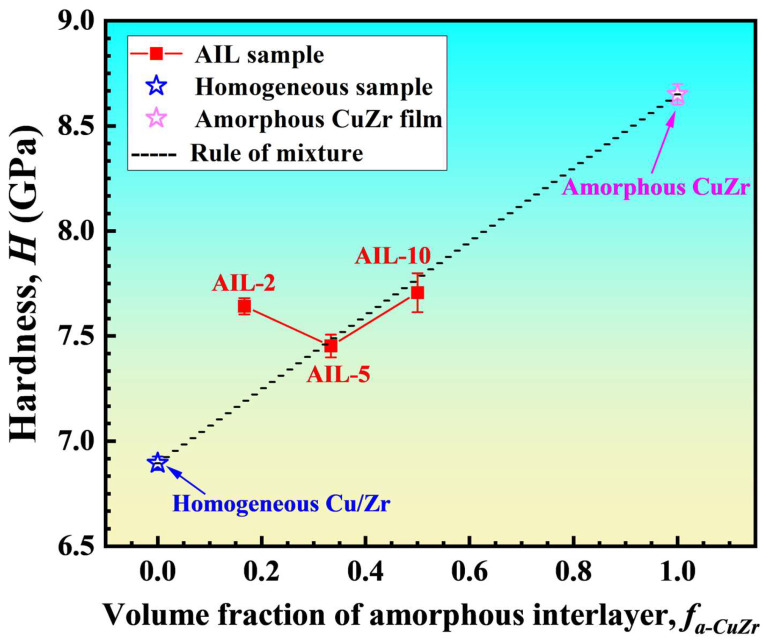
Hardness of the samples with AIL as a function of the volume fraction of AIL. The dashed lines denote the results calculated from the linear rule of mixtures.

The nano-indentation results (Table 2) show that the hardness of the Cu/Zr nanolaminates with AIL decreases first and then increases with the reduction of the AIL. As illustrated in Figure 5, all samples with AIL exhibit significantly higher hardness compared to the 10 nm homogeneous Cu/Zr samples, demonstrating the enhancement in hardness by the AIL. Considering that the Cu/Zr sample with AIL was composed of the 10 nm homogeneous Cu/Zr sample and the monolayer amorphous CuZr film, the hardness can be calculated using the rule of mixtures, as follows [49,50]:

(1) $$H_{ROM}=H_{Cu/Zr}f_{Cu/Zr}+H_{a\text{-}CuZr}f_{a\text{-}CuZr}$$

where $H_{ROM}$ is the hardness of the Cu/Zr sample with AIL, and $H_{Cu/Zr}$ and $f_{Cu/Zr}$ denote the hardness and volume fraction of the homogeneous Cu/Zr sample, respectively. $H_{a\text{-}CuZr}$ and $f_{a\text{-}CuZr}$ are the hardness and volume fraction of the monolayer amorphous CuZr film. *f_a-CuZr_* can be calculated by:

(2) $$f_{a\text{-}CuZr}=\frac{L_{AIL}}{L_{AIL}+L_{Cu/Zr}}$$

where *L_AIL_* denotes the total thickness of the AIL, and *L_Cu/Zr_* indicates the total thickness of the Cu and Zr layers in a sample. It can be seen that when the AIL thickness decreases from 10 nm to 5 nm, the measured hardness agrees well with the predictions from the rule of mixtures. Upon reducing the AIL thickness to 2 nm, the experimental hardness exceeds the prediction of the rule of mixtures, indicating a significant extra strengthening. The extremely small AIL thickness in AIL-2 nanolaminate significantly increases the density of the C/A interface, which can induce a pronounced interfacial strengthening effect. This may be responsible for the deviation of the strength of sample AIL-2 from the predictions of the rule of mixtures.

### 3.3. The Shear Instability Behavior of Cu/Zr Nanolaminates with AIL

**Figure 6:**
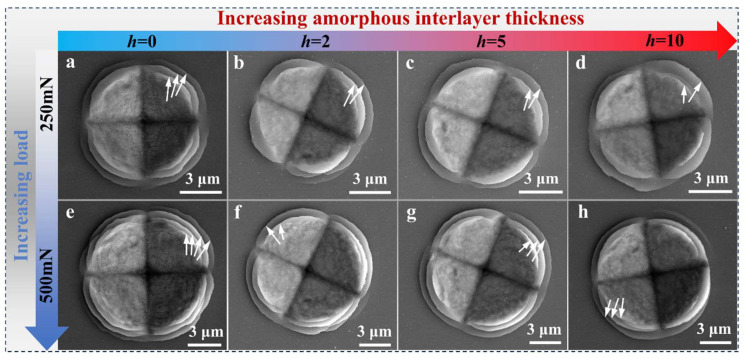
SEM images of the micro-indentation morphologies of the (**a**) homogeneous sample and AIL samples with *h* of (**b**) 2 nm, (**c**) 5 nm, and (**d**) 10 nm. (**a**–**d**) Indents with a load of 250 mN for 5 s, while (**e**–**h**) show the corresponding samples indented under a load of 500 mN for 5 s. The white arrows in (**a**–**h**) designate the SBs.

Typically, the shear banding (shear instability) of nanolaminates can be triggered by nano- and micro-indentation (equipped with a prism-shaped indenter tip) loading. With the loading process, geometric stress concentrations of the indenter tip exert a nonuniaxial stress state, causing a rotation of the layers and thereby bringing the interface into an orientation where it is loaded in shear along the maximum shear stress plane, i.e., 45° to the load axis. Thus, the tilted interface plan favors the resolved shear stress on the interface to activate the slip systems parallel to the interface and finally lead to shear banding [28,29]. Figure 6 illustrates typical SB morphologies induced by micro-indentation on the nanolaminates with AIL under 250 mN and 500 mN loads for 5 s. The indentation morphologies of the homogeneous Cu/Zr are also presented for comparison. It is evident that varying numbers of regular-shaped circular SBs emerged around the indentation surfaces of all samples, as indicated by the white arrows in Figure 6. This morphology has been widely observed in the indentation experiments of metallic multilayers [19,20,21,22,25,27]. It is widely demonstrated that the circular-shaped pileups around the residual indentation are caused by shear deformation within the nanolaminates.

**Figure 7:**
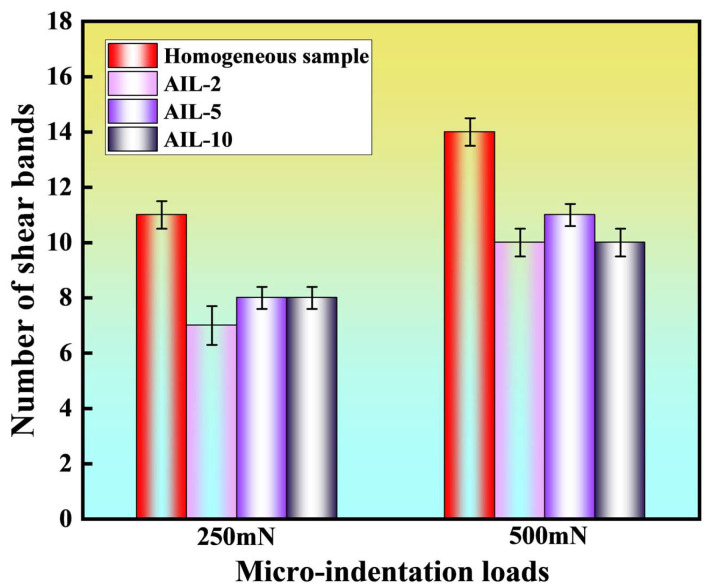
The number of SBs in the homogeneous sample and samples with AIL under loads of 250 mN and 500 mN, respectively.

Therefore, more obvious circular-shaped pileups or SBs indicate severe shear deformation within the material, corresponding to a weaker resistance to shear instability. It is clearly observed that all samples with AIL exhibit fewer SBs compared to the homogeneous Cu/Zr samples under the same indentation loading, as marked by the white arrows in Figure 6. Furthermore, the number of SBs for each sample was derived from the average value of five independent tests, while the corresponding standard deviation of the five measurements was adopted to be the error bar, as plotted in Figure 7. Notably, sample AIL-2 exhibits the fewest SBs under both indentation loads. For instance, 11 circular-shaped SBs are formed in the homogeneous sample. In contrast, the samples with AIL (samples AIL-2, AIL-5, and AIL-10) show fewer SBs as compared to the homogeneous one, with respective values of 7, 8, and 8 under a load of 250 mN.

The above micro-indentation results show that the Cu/Zr nanolaminates with AIL show stronger resistance to shear banding than the homogeneous sample, indicating that the introduction of AIL is effective in suppressing shear instability under a 3D complex stress state. Moreover, a suitable thin AIL leads to an optimal resistance to shear instability. This could be attributed to the incorporation of an AIL, which transforms the original C/C interface into the C/A interface configuration. Chen et al. [36,37] revealed that the shear instability of the 40 nm Cu/Nb nanolaminate was significantly suppressed by architecting a thick interface (composed by disordered and ordered CuNb alloy) between the Cu and Nb layers due to the strong capability of the thick interface in storing dislocations, diffusing stress concentration caused by dislocation pileup, and thus suppressing the formation of SBs. In addition, MD studies [40] have indicated that the C/A interface can act as a critical site for dislocation nucleation, emission, and annihilation during deformation, with dislocation/interface interactions improving the plastic deformation capacity of nanolaminates. In present Cu/Zr nanolaminates with AIL, the C/A interface increases the sites for dislocation nucleation during deformation, and the interaction between dislocations and the C/A interface can activate dislocation multiplication. Moreover, the C/A interface structure serves as a dislocation sink, which induces the dislocation annihilation. This mechanism of “nucleation-multiplication-annihilation” alleviates stress concentration and thus enhances the resistance to shear instability of the Cu/Zr nanolaminates.

**Figure 8:**
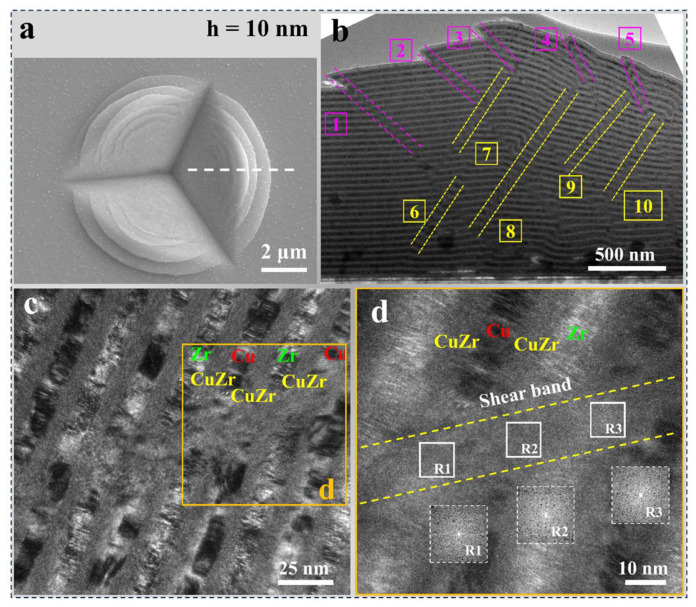
(**a**) Nano-indentation morphology of sample AIL-10. (**b**) The corresponding cross-sectional TEM images of the indented region show ten SBs. (**c**) High-magnification TEM image of the shear banding region reveals significant mixing between the adjacent crystalline and amorphous layers. (**d**) The HRTEM images of the shear banding region, as designated by the yellow box in (**c**). The insets in (**d**) are the corresponding FFTs of the core zone of the SBs, indicating that amorphous formation occurs due to the mixture of elements in constituent layers. The dashed line in (**a**) shows the position where the TEM specimen was taken.

To investigate the deformation inside the indentation, the cross-sectional microstructure of the nanolaminates with AIL (AIL-2 and AIL-10) after nano-indentation tests was characterized by TEM. The SEM image (Figure 8a) reveals multiple circular pileups in the residual nano-indentations, which is consistent with the morphology of the corresponding micro-indentation. It can be observed that ten SBs, here denoted as 1 to 10, marked by the yellow and purple dotted lines, are generated beneath the indentations of the AIL-10 samples (Figure 8b). These SBs continuously cross through the constituent layer and are distributed along the extrusion direction (1 to 5) and the indentation direction (6 to 10), respectively. It is found that the initiation and end positions of the SBs distributed along the indentation direction (6 to 10) are both within the sample. This may be attributed to the geometric constraints imposed by the Si substrate and the indenter during the formation of the SBs. In contrast, extrusion-aligned SBs (1 to 5), unconstrained by the indenter geometry, propagate freely to the sample surface. The magnified TEM image of the locally indented region, as shown in Figure 8c, shows that the modulation layer structure of the composites has been severely broken, with obvious discontinuities appearing between adjacent layers and interfaces. Moreover, the crystalline Cu and Zr layers, along with the amorphous CuZr interlayer, interpenetrate each other in the localized regions, as revealed by the HRTEM image in Figure 8d. Moreover, severe elemental mixing occurred between adjacent layers, which led to amorphization within the SB, as illustrated by the FFT image inset in Figure 8d. This indicates that severe localized deformation occurs within the composites under the shear stress. According to its characteristics, this deformation is referred to as a cutting-like shear banding.

**Figure 9:**
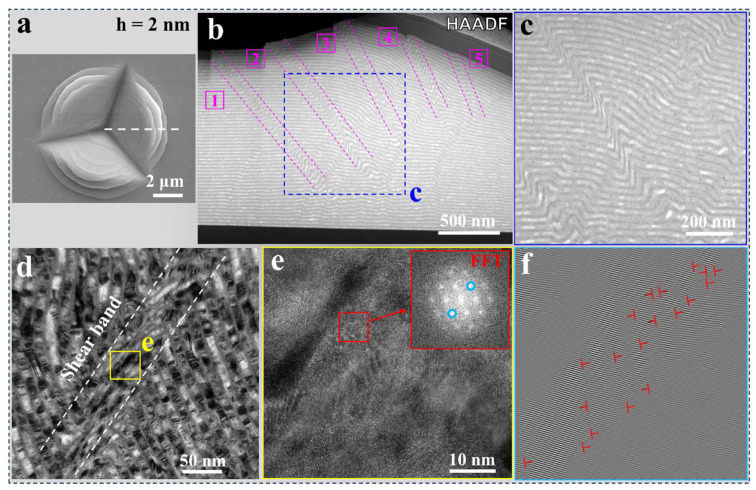
(**a**) Nano-indentation morphology of AIL-2 samples. (**b**) The corresponding cross-sectional TEM images of the indented region show five SBs. (**c**) The enlarged images of the boxed region in (**b**) and the bright-field TEM image (**d**) show the formation of the SB due to synergistic bending of multiple constituent layers. (**e**) The HRTEM images of the region as designated by the yellow box in (**d**). The FFTs inserted in (**e**) exhibit crystalline structures, confirming that constituent layers remain unaffected by shear-induced amorphization. (**f**) The IFFTs of the corresponding regions indicate a high density of dislocation distribution within the SBs. The dashed line in (**a**) indicates the position where the TEM specimen was extracted.

Figure 9 shows the cross-sectional TEM observation deformation of sample AIL-2 after nano-indentation. Although multiple circular pileups were also observed around the residual indentations of sample AIL-2 (Figure 9a), the corresponding cross-sectional TEM images (Figure 9b) reveal that only five SBs were generated internally, significantly fewer than those in the AIL-10 nanolaminate (Figure 8b). Figure 9c shows the local magnified image of the SB, indicating that the layers within the SB region are severely kinked but homogeneously shortened or elongated. Additionally, the shear region still maintains a modulated layered structure, with constituent layers and interfaces remaining continuous, as shown in Figure 9d. The FFT pattern inserted in the HRTEM image (Figure 9e) verifies that the crystalline constituent layer within the SB maintains the crystalline structure. This suggests that the AIL-2 nanolaminate undergoes more delocalized deformation compared to the AIL-10 nanolaminate, thereby exhibiting enhanced resistance to shear instability. The inverse fast Fourier transform (IFFT) results reveal, as shown in Figure 9f, a large number of dislocations along the SB direction, indicating that under shear stress, dislocation slip along the SB direction may be its primary deformation mechanism. This mode of shear deformation is referred to as kinking-like shear banding.

**Figure 10:**
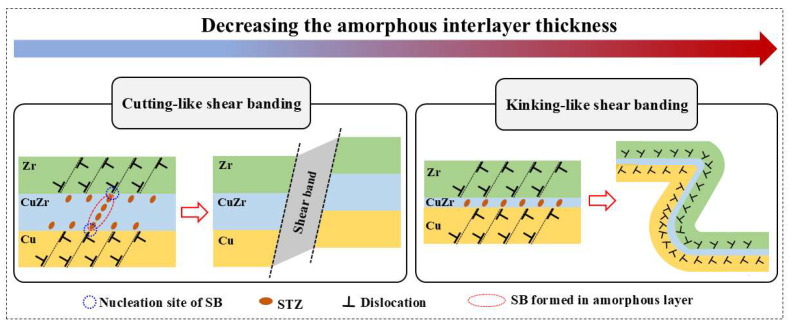
Schematic illustration of the deformation mechanism of the Cu/Zr nanolaminates with AIL.

It is worth noting that two types of SBs, i.e., cutting-like shear banding (as shown in Figure 8) and kinking-like shear banding (as shown in Figure 9), were discovered in the designed nanolaminate with AIL. This suggests that the thickness of the introduced AIL significantly modulates the shear deformation response of the Cu/Zr nanolaminates. Experiments by Cui et al. [22] also demonstrated that varying the amorphous layer thickness significantly affects the shear banding behavior of the Cu/amorphous CuZr nanolaminate. This is primarily because the plastic deformation of the amorphous phase was accommodated by the formation of SBs or STZ [51,52,53]. According to the Griffith criterion, the stress required for SB formation is described as follows [54,55]:

(3) $$\sigma_{SB}=\sqrt{\frac{2\sqrt{2}E\Gamma }{h}}$$

where Γ is the energy density per unit area of SB, *E* is Young’s modulus, and *h* is the thickness of the amorphous layer. Obviously, the stress required for the SB nucleation in the amorphous layer is increased with the decrease of the thickness of the amorphous layer. Figure 10 illustrates the formation and propagation of SBs in the Cu/Zr nanolaminate with AIL. During indentation, dislocations initiate at the C/A interface on the crystalline side and become progressively absorbed by the adjacent AIL. This absorption process subsequently triggers the activation of STZs within the amorphous layer. Due to the relatively thick AIL in the AIL-10 sample, the STZs tend to aggregate and form mature SBs within the AIL. As the SBs penetrate multiple constituent layers, they cause extensive elemental mixing between adjacent layers, forming the cutting-like shear banding (Figure 8). In sample AIL-2, the extremely thin AIL effectively suppresses the transformation of STZs into SBs. In addition, the homogeneous distribution of STZs enables deformation to be dispersed into broader amorphous regions, inducing strain delocalization. The interaction between dislocations within the crystalline layer and the C/A interface induces the rotation of the layered interface plane [29]. Upon reaching the orientation most favorable for shear, dislocations subsequently glide along the preferred shear direction in the crystalline layer (Figure 9f), resulting in the synergetic bending of the constitutive layers and the formation of kinking-like shear banding, as shown in Figure 9. In the kinking-like shear banding of the nanolaminate, plastic deformation engages a broader material volume, thereby sustaining the modulated multilayer architecture with continuous constituent layers, which can be responsible for the enhanced resistance to shear instability observed in sample AIL-2 compared to sample AIL-10.

## 4. Concluding Remarks

In summary, a novel heterogeneous Cu/Zr nanolaminate with AIL was designed and fabricated by magnetron sputtering. The effect of the introduced AIL (with thicknesses of 2 nm, 5 nm, and 10 nm, respectively) on the hardness and shear instability behavior of the nanolaminates was systematically investigated by nano- and micro-indentations. The key findings and conclusions are summarized as follows.

- The hardness of the Cu/Zr nanolaminate can be significantly elevated by the AIL, reaching 7.71 GPa in the sample with 10 nm AIL, which is 11.9% higher than the homogeneous counterpart. The hardness of the nanolaminates with AIL decreases as the thickness of the AIL is reduced from 10 nm to 5 nm, which agrees well with the predictions from the rule of mixtures; when the AIL is further reduced to 2 nm, the hardness shows abnormal increases, much higher than the predicted value according to the rule of mixtures, which was attributed to significant interface strengthening.
- Based on the indentation experiments, the shear banding of the nanolaminates was significantly suppressed by the AIL, as demonstrated by fewer SBs in samples with AIL than in the homogeneous one. Two distinct shear banding modes were observed in the nanolaminates with AIL. Specifically, a cutting-like shear banding was formed in the nanolaminate with 10 nm AIL, where the layered structure was destroyed, causing severe elemental mixing between adjacent layers and subsequent amorphization. However, a kinking-like shear banding was formed in the nanolaminate with 2 nm AIL, and synergetic bending occurred in the constitutive layers within the shear banding region. The transition from cutting-like shear banding to the kinking-like one can be attributed to the amorphous phase-dominated deformation mechanism; i.e., thicker AILs promote premature shear localization, while thinner AILs facilitate homogeneous plastic flow.

This study demonstrates that introducing an AIL with an optimal thickness in metallic nanolaminate enables higher hardness and enhanced resistance to shear instability, which provides a feasible strategy for simultaneously enhancing the strength and plasticity of metallic nanolaminates at the super-nanoscale (below 10 nm). Leveraging the inherent excellent electrical conductivity of these materials, the improved strength and plasticity of metallic nanolaminates effectively mitigate deformation damage during processing, thus elevating service life. These excellent mechanical properties make metallic nanolaminates promising candidates for various applications in microelectromechanical systems (MEMS), such as joining materials, microelectronic sensors, and flexible electronic devices.

## Figures and Tables

**Table 1:** The measured layer thicknesses of AIL-10 and AIL-2 samples.

Samples	Layer Thickness (nm)		
	Cu	Zr	Amorphous CuZr
AIL-10	9.63 ± 0.54	9.32 ± 0.49	10.12 ± 0.43
AIL-2	10.15 ± 0.91	8.61 ± 0.56	2.98 ± 0.41

**Table 2:** Nano-indentation-derived hardness (*H*) and elastic modulus (*E*) of the nanolaminates with AIL. Those of the homogeneous Cu/Zr nanolaminates and amorphous CuZr films are also included.

Samples	Nano-Hardness, *H* (GPa)	Elastic Modulus, *E* (GPa)
AIL-10	7.71 ± 0.09	138 ± 2
AIL-5	7.45 ± 0.05	135 ± 2
AIL-2	7.64 ± 0.04	137 ± 3
Homogeneous	6.89 ± 0.03	145 ± 1
CuZr film	8.65 ± 0.05	136 ± 2

## Data Availability

All data generated or analyzed during this study are included in this published article or are available from the corresponding authors upon reasonable request.
